# Hydrogen Sulfide Prevents Sleep Deprivation-Induced Hippocampal Damage by Upregulation of Sirt1 in the Hippocampus

**DOI:** 10.3389/fnins.2020.00169

**Published:** 2020-03-11

**Authors:** Jin-Xi Zuo, Min Li, Li Jiang, Fang Lan, Yi-Yun Tang, Xuan Kang, Wei Zou, Chun-Yan Wang, Ping Zhang, Xiao-Qing Tang

**Affiliations:** ^1^Department of Neurology, Affiliated Nanhua Hospital, University of South China, Hengyang, China; ^2^Department of Neurology, The First Affiliated Hospital, University of South China, Hengyang, China; ^3^Institute of Neurology, The First Affiliated Hospital, University of South China, Hengyang, China; ^4^Department of Physiology, Hengyang Medical College, University of South China, Hengyang, China

**Keywords:** sleep deprivation, hydrogen sulfide, hippocampal damage, silence information regulating factor 1, oxidative stress

## Abstract

Sleep deprivation (SD) induces hippocampal damage. Hydrogen sulfide (H_2_S) is a neuronal protective factor. Silence information regulating factor 1 (Sirt1) plays an important role in neuroprotection. Therefore, this study was aimed at exploring whether H_2_S meliorates SD-induced hippocampal damage and whether Sirt1 mediates this protective role of H_2_S. We found that sodium hydrosulfide (NaHS, a donor of H_2_S) alleviated SD-generated hippocampal oxidative stress, including increases in the activation of SOD and the level of GSH as well as a decrease in the level of MDA. Meanwhile, we found that NaHS reduced SD-exerted hippocampal endoplasmic reticulum (ER) Stress, including downregulations of GRP78, CHOP, and cleaved-caspase-12 expression. Moreover, NaHS reduced the apoptosis in the SD-exposed hippocampus, and this included decreases in the number of apoptotic cells and the activation of caspase-3, downregulation of Bax expression, and upregulation of Bcl-2 expression. NaHS upregulated the expression of Sirt1 in the hippocampus of SD-exposed rats. Furthermore, Sirtinol, the inhibitor of Sirt1, abrogated the protection of NaHS against SD-exerted hippocampal oxidative stress, ER stress, and apoptosis. These results suggested that H_2_S alleviates SD-induced hippocampal damage by upregulation of hippocampal Sirt1.

## Introduction

Sleep is essential to the balance of human physical health. It has been shown that most adults do not get enough sleep (Centers for Disease and Prevention, 2011). Sleep deprivation (SD) causes physiological, psychological, and behavioral change (Walker and Stickgold, 2006; Ohayon et al., 2010; Dai et al., 2012), and it especially has an effect on the central nervous system (CNS) (Nabaee et al., 2018). In recent years, the effects of SD on cognitive function have attracted wide attention (Killgore, 2010; Kaliyaperumal et al., 2017; Nasehi et al., 2018). However, the underlying mechanisms of these effects remain unclear. The hippocampus, as a key component of the limbic systems, plays an important role in the regulation of cognition (Shohamy and Turk-Browne, 2013). The important role of the hippocampus for cognitive functions is well established (Squire and Zola, 1996; Eichenbaum, 2000). It has been demonstrated that paradoxical SD increases the hippocampal oxidative stress in rats (Singh et al., 2008). Moreover, SD increases endoplasmic reticulum (ER) stress (Hakim et al., 2015) and eventually leads to neuronal apoptosis in the brain (Qiu et al., 2016). In addition, SD causes hippocampal synaptic and membrane excitability changes, which indicated that SD determinately affects hippocampus damage (McDermott et al., 2003). Therefore, it is of great academic value to explore the potential therapeutic approaches for hippocampal damage, which may provide a new target for the treatment of SD-induced cognitive impairment.

Hydrogen sulfide (H_2_S), the third gasotransmitter after carbon monoxide (CO) and nitric oxide (NO) (Wang, 2010), exerts protective effect in the CNS (Zhang and Bian, 2014; Panthi et al., 2016; Elbassuoni and Nazmy, 2018). Emerging evidence has indicated that H_2_S has anti-oxidative, anti-ER stress, and antiapoptotic properties in the CNS (Jiang et al., 2011; Nie et al., 2013; Li et al., 2014; Zhang et al., 2014). Of note, our groups have demonstrated the protective effect of H_2_S against chronic unpredictable mild stress-induced oxidative stress and ER stress in the hippocampus (Hu et al., 2016; Liu et al., 2017). We previously provided evidence that H_2_S inhibits neuronal apoptosis in the hippocampus of homocysteine-induced rats (Wei et al., 2014). These data suggested that H_2_S plays an important role in counteracting hippocampal damage. Thus, we speculated that H_2_S ameliorates oxidative stress, ER stress, and apoptosis in the hippocampus of SD-treated rats.

Silence information regulating factor 1 (Sirt1), an NAD^+^ dependent class III histone deacetylases (Imai et al., 2000; Smith et al., 2000; Cohen et al., 2004), plays important roles in neuroprotection and neuroregulation (Han, 2009; Zhang et al., 2011; Donmez, 2012). It has been demonstrated that SD decreases the expression of Sirt1 in the hippocampus (Chang et al., 2009) and that Sirt1 deficiency induces hippocampal damage (Kim et al., 2018). Moreover, Sirt1 activation relieves ischemia-induced and D-galactose-induced hippocampal apoptosis (Meng et al., 2015; Wu et al., 2015). Furthermore, our previous study testified that H_2_S upregulates the expression of Sirt-1 in the hippocampus (Liu et al., 2017; Tang et al., 2018) and that H_2_S inhibits chronic restrain stress (CRS)-induced hippocampal damage in rats via upregulating the expression of Sirt-1 (Li et al., 2017). Therefore, we speculated that the protection of H_2_S against SD-induced hippocampal damage is also via upregulating hippocampal Sirt1.

In the present work, we demonstrated the suppressive effects of H_2_S on hippocampal oxidative Stress, ER stress, and apoptosis in SD-exposed rats. We also proved that H_2_S significantly upregulated Sirt1 expression in the hippocampus of SD-exposed rats. Furthermore, Sirtinol, a Sirt1 inhibitor, reversed the protective effects of H_2_S against SD-induced hippocampal oxidative Stress, ER stress, and apoptosis. Taken together, we identified a critical role of H_2_S on the protection against SD-induced hippocampal damage and the mediatory role of hippocampal Sirt1 in the protection of H_2_S against SD-induced hippocampal damage.

## Materials and Methods

### Reagents

Sodium hydrosulfide (NaHS, a donor of H_2_S), Sirtinol (a Specific Sirt-1 inhibitor), and Cleaved caspase-12-antibody were obtained from Sigma-Aldrich (St Louis, MO, United States). Pelltobarbitalum Natricum was bought from Germany. The Specific monoclonal antibodies of CPR78, CHOP, Bax Bcl2, and Sirt1 were purchased from Abcam (Hong Kong, China). The malondialdehyde (MDA) assay kit, superoxide dismutase (SOD) assay kit, glutathione (GSH) enzyme-linked immunosorbentassay (ELISA) kits, and caspase-3 Elisa Kit were purchased from USCN Life ScienceInc (Wuhan, Hubei, China).

### Animals

The adult male Wistar rats (200 g∼220 g) were purchased from the SJA Lab Animal Center of Changsha (Changsha, Hunan, China). The rats were fed within single cage at a temperature of (25 ± 2)°C, constant humidity, natural illumination, and good ventilation. The rats were free to eat and drink, and the padding was changed regularly (two times a week). The subjects were touched for 3 min every day to eliminate the strangeness with the testers and to reduce the interference and influence of human factors and surrounding environment on the experimental rats. All the procedures and animal experiments were strictly conducted according to the National Institutes of Health Guide for the Care and Use of Laboratory Animals and were approved by the Animal Use and Protection Committee of University of South China.

#### Induction of Sleep Deprivation

The sleep deprivation model was established by the modified multiple platform method (MMPM) (Suchecki and Tufik, 2000). Animals were placed in a water tank (170 cm × 70 cm × 50 cm) that was filled with 19 small platforms (diameter 6.5 cm) surrounded by water (24 ± 1°C), and the horizontal plane was 1 cm below the small platform. The model was used to hinder the total sleep (especially REM sleep). When the rats entered REM sleep, muscle atonia caused animals to hit the water and wake up, leading to sleep deprivation (Machado et al., 2004). Rats were exposed to sleep deprivation for 72 h on the platform.

### Drug Treatments and Experimental Schedule

After adaptation for 7 days, all rats were randomly divided into seven treatment groups: in the control group, rats were injected with PBS (i.p.) for 10 days; in the SD group, rats were exposed to the modified multiple platform method (MMPM) for 72 h; in the SD + NaHS (30 μmol/kg or 100 μmol/kg) group, rats were pretreated with NaHS (30 or 100 μmol/kg/d, ip) for 7days and then cotreated with SD for 72 h; in the SD + NaHS (100 μmol/kg) + Sirtinol (10 nm, i.c.v.) group, rats were pretreated with NaHS (100 μmol/kg/d, ip) for 7 days, and then cotreated with SD for 72 h and Sirtinol (10 nmol/d, icv) for 3 days; and in the NaHS (100 μmol/kg) alone group and Sirtinol alone group, rats were injected with NaHS (100 μmol/kg, 10 days, i.p.), and Sirtinol (10 nmol, 10 d, i.c.v.). NaHS was dissolved in Phosphate Buffered Saline (PBS), and Sirtinol (5 mg) was diluted in sterilized artificial CSF (ACSF)/0.5% DMSO. All rats were killed, and the hippocampi were rapidly collected and stored at −80°C for analysis.

### Intracerebroventricular Injection

Rats were deeply anesthetized with 1% sodium pentobarbital (45 mg/kg, i.p.) and were then fixed on a stereotaxic apparatus for operation. The area around the incision was clipped by sterile surgical scissors. Sirtinol (10 nmol) was injected into the unilateral lateral ventricle using coordinates—AP: 1.0 mm, R or L: 1.5 mm—with an injection rate of 0.75 μL/min using a 10-μL Hamilton syringe. In order to guarantee the entire injection had been completely delivered, the needle was gradually pulled out halfway and kept in cannula for an extra 2 min before being removed. After the surgery, all rats were injected subcutaneously with administered penicillin (q.d.) for three consecutive days.

### Biochemical Analysis for MDA, GSH, Caspase-3, and SOD

The hippocampus tissues were homogenized (10% w/v) with 0.1 mol/L of PBS and then centrifuged at 12,000 × *g* for 10 min. The supernatants were collected, and total protein concentrations were quantified using a BCA Protein Assay. The levels of MDA, GSH, and Caspase-3 were analyzed by ELISA kits. The activity of SOD was measured by the NBT assay kit. Specific steps were laid out according to the manufacturer’s instruction on the reagent kits.

### Western Blot Detection for the Expressions of CPR78, CHOP, Cleaved Caspase-12, Bax, Bcl-2, and Sirt1 in the Hippocampus Tissue

Hippocampal tissue was removed and homogenized in an ice-cold homogenizing buffer (20 mM Tris-Cl, pH 7.4, 150 mM NaCl, 1 mM EDTA, 1% Triton X-100, 1 mM PMSF). After centrifugation at 12,000 × g for 30 min at 4°C, the supernatant was collected, and the protein content was subsequently assayed by using a BCA Protein Assay Kit (Beyotime, Shanghai, China). The protein was then diluted by PBS to the same concentration. The protein extract with an equivalent volume for each sample was run on sodium dodecyl sulfate-polyacrylamide gel electrophoresis. After that, the protein was transferred to a PVDF membrane using a wet transfer system and blocked with TBST (50 mmol/L Tris–HCl, pH 7.5,150 mmol/L NaCl, 0.1% Tween-20) for 2 h at room temperature. Then the PVDF membranes were irrespectively incubated with primary antibodies against GPR78, CHOP, Cleaved Caspase-12, Bcl-2, Bax, Sirt1 (1:1000), and β-actin (1:2000) overnight at 4°C. Next day, the membrane was washed three times with TBST (50 mmol/L Tris–HCl, pH 7.5,150 mmol/L NaCl, 0.1% Tween-20) for 15 min then incubated with secondary antibody (1:5000) for 2 h. Finally, Protein bands were analyzed through a developing system equipped with a software BIO-ID (Vilber Lourmat, France).

### Tunel Staining

TUNEL staining was put into effect using an Apoptag Peroxidase *In Situ* Apoptosis Detection Kit S7100 (EMD Millipore, Billerica, MA, United States) on the basis of the manufacturer’s instructions. The cerebral sections of the CA3 area of hippocampus were incubated with the TUNEL reaction mixture at 37°C for 60 min, and they were also incubated with a proteinase K solution for 15 min at 37°C to enhance permeability, and then the sections were put into the 3% hydrogen peroxide solution prepared with methanol to block the endoperoxidase. Finally, they were treated with a DAB-substrate solution. Five hippocampus sections of each rat were selected. The sections of the CA3 area images were acquired using a fluorescent microscope (Nikon, Japan), and the number of positive TUNEL cells were counted with Image J and Image-Pro Plus 6.0.

### Statistical Analysis

The statistical analysis of all data was carried out with SPSS 20.0 software. The experimental data were displayed as the mean ± standard error of the mean. The significance of intergroup differences was analyzed by one-way ANOVA and the least significant difference (LSD) *post hoc* test. The level of significance was considered at *P* < 0.05.

## Results

### H_2_S Inhibites SD-Generated Hippocampal Oxidative Stress

**FIGURE 1:**
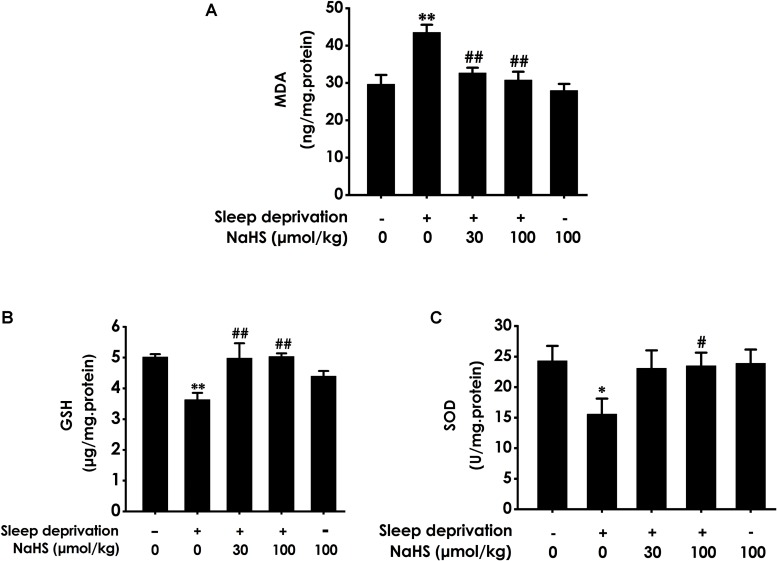
Effect of hydrogen sulfide on sleep deprivation-exerted hippocampal oxidative stress in rats. Rats were pretreated with NaHS (30 and 100 μmol/kg/d, ip) for 7 days and then cotreated with SD for 72 h. The level of MDA **(A)** and the content of GSH **(B)** in hippocampus were detected by ELISA kit. The activity of SOD **(C)** was measured by the NBT assay kit. Values are the means ± SEM (*n* = 3). ^∗^*P* < 0.05, ^∗∗^*P* < 0.01, vs. control; ^#^*P* < 0.05, ^##^*P* < 0.01, vs. SD-treated alone group.

To investigate whether H_2_S inhibits SD-generated hippocampal oxidative stress, we explored the effects of H_2_S on the generation of MDA and GSH as well as the activity of SOD in the hippocampus of rats exposed to SD. After exposure with SD, the content of MDA (Figure 1A) in the hippocampus was significantly increased, while the level of GSH (Figure 1B) and the activity of SOD (Figure 1C) in the hippocampus were significantly decreased. These data indicated that SD induced hippocampal oxidative stress. However, treatment with NaHS (30 and 100 μmol/kg) significantly decreased the content of MDA (Figure 1A) as well as markedly increased the level of GSH (Figure 1B) and the activity of SOD (Figure 1C) in the hippocampus of SD-treated rats. NaHS (100 μmol/kg) alone had no effect on the content of MDA, the level of GSH, and the activity of SOD. Taken together, these results indicated that H_2_S prevents SD-induced hippocampal oxidative stress.

### H_2_S Prevents SD-Induced Hippocampal ER Stress

**FIGURE 2:**
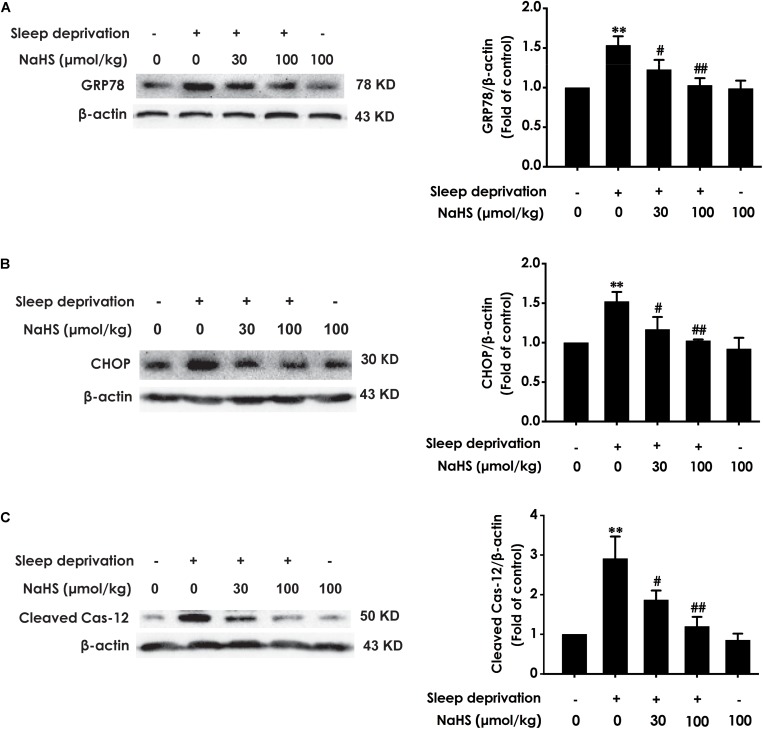
Effect of hydrogen sulfide on sleep deprivation-induced hippocampal ER stress in rats. Rats were pretreated with NaHS (30 and 100 μmol/kg/d, ip) for 7 days and then cotreated with SD for 72 h. The expression of CPR78 **(A)**, CHOP **(B)**, and cleaved caspase-12 **(C)** were detected by western blotting. In all blots, β-actin was used to determine control. Values are the means ± SEM (*n* = 3). ^∗∗^*P* < 0.01, vs. control; ^#^*P* < 0.05,^##^*P* < 0.01, vs. SD-treated alone group.

To investigate whether H_2_S prevents SD-induced hippocampal ER stress, we explored the effect of H_2_S on the expressions of GPR78, CHOP, and cleaved caspase-12 in the hippocampus of SD-exposed rats. The expressions of GRP78 (Figure 2A), CHOP (Figure 2B), and cleaved caspase-12 (Figure 2C) were significantly increased in the hippocampus of SD-treated rats, which indicated that SD induces hippocampal ER stress. However, treatment with NaHS (30 and 100 μmol/kg) observably decreased the expressions of GRP78 (Figure 2A), CHOP (Figure 2B), and cleaved caspase-12 (Figure 2C) in the hippocampus of SD-exposed rats. NaHS (100 μmol/kg) alone had no effect on the expressions of GRP78, CHOP, and cleaved caspase-12 proteins. These results indicated that H_2_S prevents SD-induced hippocampal ER stress.

### H_2_S Attenuates SD-Exerted Hippocampal Apoptosis

**FIGURE 3:**
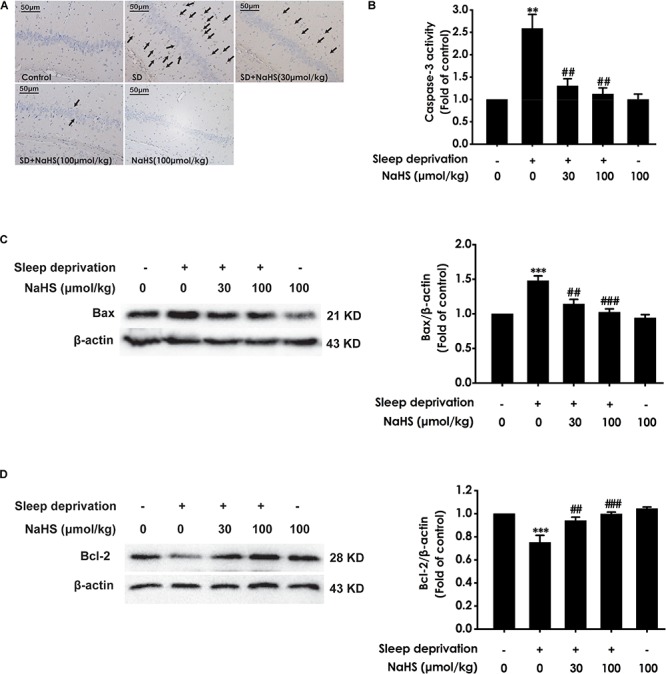
Effect of hydrogen sulfide on sleep deprivation-induced hippocampal apoptosis in rats. Rats were pretreated with NaHS (30 and 100 μmol/kg/d, ip) for 7 days and then cotreated with SD for 72 h. The apoptotic cells in the CA3 area of hippocampus **(A)** were assessed using Tunel staining, and the arrows indicate the apoptotic cells. The activity of caspase-3 **(B)** in the hippocampus was detected by ELISA kits. The expressions of Bax **(C)** and Bcl-2 **(D)** in the hippocampus were detected by western blot using anti-Bax antibody and anti-Bcl-2 antibody, respectively. In all blots, β-actin was used to determine control. Values are the mean ± S.E.M. (*n* = 3–4), ^∗∗^*P* < 0.01, ^∗∗∗^*P* < 0.001 vs. control group. ^##^*P* < 0.01, ^###^*P* < 0.001, vs. SD-treated alone group.

Next, we also investigated whether H_2_S inhibits SD-induced hippocampal apoptosis. The number of apoptotic cells (Figure 3A) in the hippocampal CA3 region of SD-exposed rats was markedly increased. In addition, in the hippocampus of SD-exposed rats, the activity of caspase-3 was enhanced (Figure 3B), and the expression of Bax (Figure 3C) was significantly upregulated, while the expression of Bcl-2 (Figure 3D) was downregulated. These data indicated that SD induces hippocampal apoptosis. However, treatment with NaHS (30 and 100 μmol/kg) significantly decreased the number of apoptotic cells in the hippocampal CA3 region (Figure 3A) of SD-exposed rats and reversed SD-exerted activation of caspase-3 (Figure 3B), upregulation of Bax expression (Figure 3C), and downregulation of Bcl-2 expression (Figure 3D) in the hippocampus. NaHS (100 μmol/kg) alone had no effect on the number of apoptotic cells in the hippocampal CA3 region, the activity of caspase-3, and the expressions of Bax and Bcl-2. These results indicated the protection of H_2_S against SD-induced hippocampal apoptosis.

### H_2_S Upregulates Hippocampal Sirt1 Expression in SD-Exposed Rats

**FIGURE 4:**
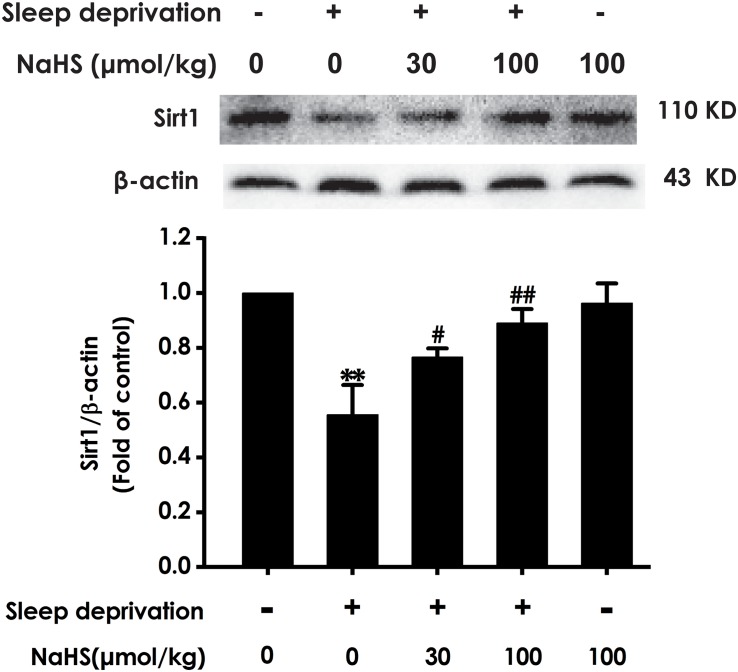
Effect of hydrogen sulfide on the level of hippocampal Sirt1 in sleep deprivation-exposed rats. Rats were pretreated with NaHS (30 and 100 μmol/kg/d, ip) for 7 days and then cotreated with SD for 72 h. The expression of hippocampal Sirt1 was measured by western blotting. Values are the means ± SEM (*n* = 3–4). ^∗∗^*P* < 0.01, vs. control; ^#^*P* < 0.05, ^##^*P* < 0.01, vs. SD-treated alone group.

To determine whether Sirt1 mediates the protective effect of H_2_S against SD-induced hippocampal damage, we explored the effect of H_2_S on the expression of Sirt1 protein in the hippocampus of SD-exposed rats. After exposure with SD, the expression of Sirt-1 in the hippocampus of rats was markedly decreased (Figure 4). However, treatment with NaHS (30 and 100 μmol/kg) observably increased the expression of Sirt1 in hippocampus of SD-exposed rats (Figure 4). These results indicated that H_2_S upregulates the expression of hippocampal Sirt1 in SD-exposed rats.

### Inhibition of Sirt-1 by Sirtinol Attenuates the Protection of H_2_S Against SD-Exerted Hippocampal Oxidative Stress

**FIGURE 5:**
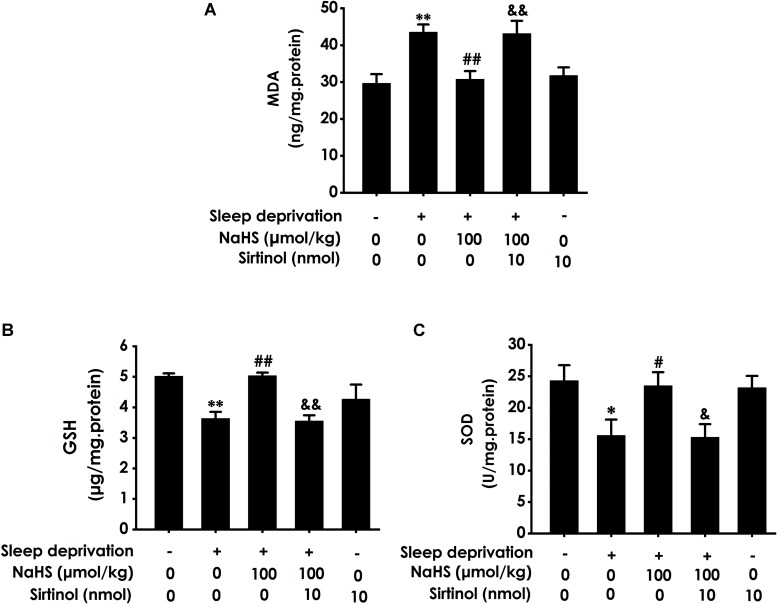
Effect of Sirtinol on hydrogen sulfide ameliorated hippocampal oxidative stress in sleep deprivation-exposed rats. Rats were pretreated with NaHS (100 μmol/kg/d, ip) for 7 days and then cotreated with SD and Sirtinol (10 nmol/d, icv) for 72 h. The levels of MDA **(A)** and GSH **(B)** in the hippocampus were detected by ELISA kit. The activity of SOD **(C)** in the hippocampus was measured by the NBT assay kit. Values are the means ± SEM (*n* = 3). ^∗^*P* < 0.05, ^∗∗^*P* < 0.01, vs. control; ^#^*P* < 0.05, ^##^*P* < 0.01, vs. SD-treated alone group; ^&^*P* < 0.05, ^&⁣&^*P* < 0.01, vs. cotreated with SD and NaHS (100 μmol/kg/d, i.p.) group.

To confirm the mediatory role of Sirt1 in the protection of H_2_S against SD-induced hippocampal damage, we first explored whether Sirtinol, a specific Sirt1 inhibitor, blocks the protection of H_2_S against SD-induced hippocampal oxidative stress. Sirtinol (10 nmol × 1w, i.c.v.) increased the hippocampal MDA level (Figure 5A) and decreased the hippocampal GSH content (Figure 5B) as well as SOD activity (Figure 5C) in the rats cotreated with SD and NaHS (100 μmol/kg/d, ip), which indicated that Sirtinol reverses the antagonistic effect of H_2_S on SD-induced hippocampal oxidative stress in rats.

### Inhibition of Sirt-1 by Sirtinol Reverses the Meliorating Effect of H_2_S on SD-Exerted Hippocampal ER Stress

**FIGURE 6:**
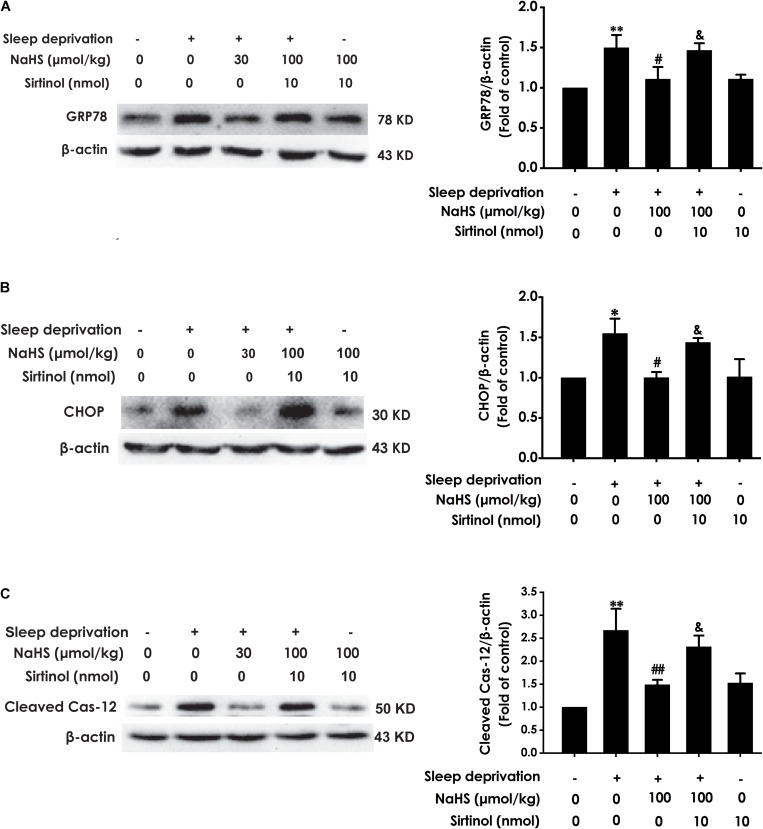
Effect of Sirtinol on hydrogen sulfide ameliorated hippocampal ER stress in sleep deprivation-exposed rats. Rats were pretreated with NaHS (100 μmol/kg/d, ip) for 7 days and then cotreated with SD and Sirtinol (10 nmol/d, icv) for 72 h. The expression of CPR78 **(A)**, CHOP **(B)**, and cleaved caspase-12 **(C)** in the hippocampus were detected by western blotting. In all blots, β-actin was used to determine control. Values are the means ± SEM (*n* = 3). ^∗^*P* < 0.05, ^∗∗^*P* < 0.01, vs. control; ^#^*P* < 0.05, ^##^*P* < 0.01, vs. SD-treated alone group; ^&^*P* < 0.05, vs. cotreated with NaHS (100 μmol/kg/d, i.p.) and SD treated group.

Next, we investigated whether Sirtinol reverses the protective role of H_2_S on SD-induced ER stress. Sirtinol (10 nmol, i.c.v.) significantly increased the expressions of GPR78 (Figure 6A), CHOP (Figure 6B), and cleaved caspase-12 (Figure 6C) protein in the hippocampus of rats cotreated with SD and NaHS (100 μmol/kg/d, i.p.), which indicated that Sirtinol abrogates the protective action of H_2_S on SD-induced hippocampal ER stress.

### Inhibition of Sirt-1 by Sirtinol Antagonizes H_2_S-Ameliorated Hippocampal Apoptosis in SD-Exposed Rats

**FIGURE 7:**
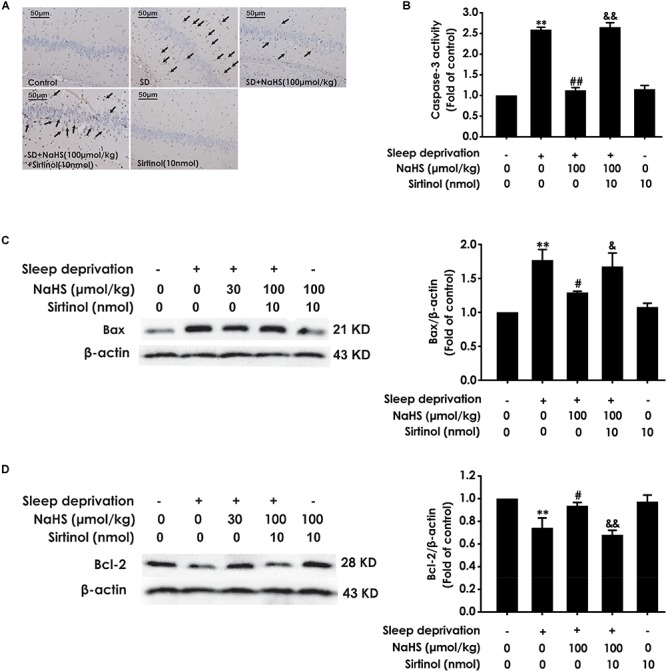
Effect of Sirtinol on hydrogen sulfide ameliorated hippocampal apoptosis in sleep deprivation-induced rats. Rats were pretreated with NaHS (100 μmol/kg/d, ip) for 7 days and then cotreated with SD and Sirtinol (10 nmol/d, icv) for 72 h. The apoptotic cells in the CA3 area of hippocampus **(A)** were assessed using Tunel staining and the arrow indicated the apoptotic cell. The activity of caspase-3 **(B)** in the hippocampus was detected by ELISA kits. The expressions of Bax **(C)** and Bcl-2 **(D)** in the hippocampus were measured by western blotting. In all blots, β-actin was used to determine control. Values are the mean ± S.E.M. (*n* = 3). ^∗∗^*P* < 0.01, vs. control group; ^#^*P* < 0.05, ^##^*P* < 0.01, vs. SD-treated alone group; ^&^*P* < 0.05, ^&⁣&^*P* < 0.01, vs. cotreated with NaHS (100 μmol/kg/d, i.p.) and SD treated group.

We also detected whether Sirtinol blocks the protective role of H_2_S in SD-induced hippocampal apoptosis. Sirtinol (10 nmol, i.c.v.) remarkably increased the amount of apoptotic cells in the hippocampal CA3 region (Figure 7A), enhanced the activity of caspase-3 (Figure 7B), and upregulated the expression of Bax (Figure 7C), while it also significantly downregulated the expression of Bcl-2 (Figure 7D) in the hippocampus of rats cotreated with SD and NaHS (100 μmol/kg/d, i.p.), which indicated that Sirtinol abrogates the protective action of H_2_S in SD-induced hippocampal apoptosis.

## Discussion

In the present work, we investigated whether H_2_S ameliorates SD-induced hippocampal damage and whether Sirt1 mediates this role of H_2_S. There were several major findings: (1) *NaHS* (a donor of H_2_S) mitigates SD-induced hippocampal oxidative stress, ER stress, and apoptosis in SD-exposed rats; (2) NaHS upregulates the expression of Sirt1 in the hippocampus of SD-generated rats; and (3) Sirtinol, an inhibitor of Sirt1, abolishes the protection of H_2_S against hippocampal oxidative stress, ER stress, and apoptosis in SD-induced rats. Taken together, these data indicated that H_2_S alleviates SD-induced hippocampal damage, which is via upregulation of hippocampal Sirt1.

Sleep deprivation is an increasingly serious disease in the world, which detrimentally effects on cognitive function (Basner et al., 2007). Increasing evidence in SD animal models also demonstrated that SD induces cognitive impairment (Zhao et al., 2017). In addition, it has been shown that SD results in hippocampal damage (Havekes and Abel, 2017), which is the key link of cognitive dysfunction (Jia et al., 2015). Therefore, antagonizing hippocampal damage may be a therapeutic approach for SD-induced cognitive dysfunction. H_2_S, as an important neuromodulator and neuroprotectant (Kimura, 2002; Hu et al., 2011; Kimura et al., 2012), plays an essential role in alleviating hippocampal damage (Alzoubi et al., 2018). It has been demonstrated that exogenous H_2_S alleviates oxidative stress-induced rat hippocampal damage (Jiang et al., 2011) and that H_2_S prevents cognitive dysfunction via ameliorating hippocampal damage (Zou et al., 2017). Therefore, it is necessary to explore the potential effect of H_2_S on SD-induced hippocampal damage.

In the present work, rats were pretreated with NaHS (a donor of H_2_S) for 7 days cotreated with SD for 72 h and the levels of oxidative stress, ER stress and apoptosis in hippocampal were detected. We found that NaHS decreased the level of MDA, increased the activation of SOD, and raised the level of GSH in the hippocampus of SD-exposed rats, which indicated that H_2_S protects against SD-generated hippocampal oxidative stress. Meanwhile, these data are consistent with the previous study that H_2_S decreased the level of MDA, increased the activation of SOD, and raised the level of GSH in the hippocampus of rats undergoing heroin withdrawal (Jiang et al., 2011), Moreover, we found that H_2_S downregulated the expressions of ER stress-related proteins, including GRP78, CHOP and cleaved-caspase-12, in the hippocampus of SD-induced rats. GRP78, CHOP, and caspase-12 are three important markers of ER stress (Hetz, 2012). Our results indicated the protective effects of H_2_S against SD-induced hippocampal ER stress. This finding was consistent with the previous observations that H_2_S prevents the hippocampal ER Stress in chronic unpredictable mild stress-exposed rats (Liu et al., 2017). It has been found that H_2_S attenuates isoflurane-induced hippocampal apoptosis (Hu et al., 2017). Therefore, we investigated whether H_2_S attenuates SD-induced apoptosis in hippocampus. Bax is a apoptotic protein, Bcl-2 is an anti-apoptotic protein, and Caspase-3 is a critical executioner of apoptosis (Konstantinidou et al., 2007; Jiang et al., 2011). Notably, we also found that NaHS decreased the number of apoptotic cells in the hippocampal CA3 region, suppressed the activity of caspase-3, downregulated the expression of Bax, and upregulated the expression of Bcl2 in the hippocampus of SD-exposed rats, which indicated that H_2_S attenuates the hippocampal apoptosis in SD-induced rats. Similarity, it has been found that the activity of caspase-3 was decreased, the expression of Bax protein was upregulated, and that the expression of Bcl-2 protein was downregulated in the hippocampus of rats undergoing heroin withdrawal, which were reversed by treatment with exogenous H_2_S (Jiang et al., 2011). Taken together, these data suggested that H_2_S prevents hippocampal damage evoked by SD. It has been confirmed that the protection of H_2_S on recurrent febrile seizures-induced hippocampal damage (Han et al., 2005), which offered a reasonable explanation for our present results.

Next, we further explored the underlying mechanisms for the protection of H_2_S against SD-induced hippocampal damage. Sirt1, is a member of the sirtuin family of nuclear and cytoplasmic class III histone deacetylases (Sauve et al., 2006), which are expressed in the hippocampus (Michan et al., 2010). Accumulating evidence demonstrated that Sirt1 plays an important role for neuroprotection in several models of neurodegenerative diseases, such as Alzheimer’s disease (AD) (Fujita and Yamashita, 2018), while the level of hippocampal Sirt1 were significantly decreased in patients with AD (Cao et al., 2018). Meanwhile, it has been demonstrated that oxymatrine attenuated hippocampus ischemia/reperfusion injury through upregulation of Sirt1 (Zhou et al., 2018), indicating that Sirt1 acts as a key factor in hippocampal damage. In addition, SD decreases the expression of Sirt1 in hippocampus (Chang et al., 2009). Does Sirt1 mediate the protection of H_2_S against SD-induced hippocampal damage? Interestingly, our previous study testified that H_2_S upregulates the expression of Sirt1 in hippocampus (Tang et al., 2018) and that Sirt1 mediates the protection of H_2_S against chronic restrain stress-induced hippocampal damage in rats (Li et al., 2017). Therefore, the speculation that Sirt1 mediates the protection of H_2_S against SD-induced hippocampus damage is reasonable. Our present work showed that SD caused decreases in the expression of Sirt1 in hippocampus and that H_2_S observably increased the expression of Sirt1 in the hippocampus of the SD-generated damage. Moreover, Sirtinol, the Sirt1 inhibitor, blocked the protective effect of H_2_S on SD-induced hippocampal oxidative stress, ER stress, and apoptosis in rats. This finding is consistent with the previous report that Sirt1 reverses the protective effect of H_2_S on hippocampal damage in chronic restrain stress-induced rats (Li et al., 2017). These data indicated that upregulating the expression of Sirt1 in hippocampus mediates the protective role of H_2_S in SD-induced hippocampal damage.

In summary, the present study elucidated the protective effect of H_2_S on SD-induced hippocampal damage, which is mediated by the upregulation of Sirt1 in hippocampus. Our finding suggested that H_2_S may act a novel potential therapeutic agent for SD-induced hippocampal damage, which opens a new perspective for the prevention and treatment of cognitive dysfunction after SD.

## Data Availability Statement

All datasets generated for this study are included in the article/supplementary material.

## Ethics Statement

The animal study was reviewed and approved by the Animal Use and Protection Committee of University of South China.

## Author Contributions

X-QT and WZ contributed conception and design of the study. X-QT, PZ, and C-YW guided and supervised the study. J-XZ, ML, LJ, FL, Y-YT, and XK performed the experiments. J-XZ, ML, and LJ analyzed the data of the experiments and wrote the manuscript. All authors contributed to manuscript revision and read and approved the submitted version.

## Conflict of Interest

The authors declare that the research was conducted in the absence of any commercial or financial relationships that could be construed as a potential conflict of interest.
